# Incidence of Dysmagnesemia among Medically Hospitalized Patients and Associated Clinical Characteristics: A Prospective Cohort Study

**DOI:** 10.1155/2023/6650620

**Published:** 2023-10-04

**Authors:** Zahra Al Shukri, Juhaina Salim Al-Maqbali, Abdullah M. Al Alawi, Nafila Al Riyami, Sulaiman Al Riyami, Hiba Al Alawi, Qatiba Al Farai, Henrik Falhammar

**Affiliations:** ^1^Internal Medicine Residency Training Program, Oman Medical Specialty Board, Muscat, Oman; ^2^Department of Pharmacology and Clinical Pharmacy, College of Medicine and Health Science, Sultan Qaboos University, Muscat, Oman; ^3^Department of Pharmacy, Sultan Qaboos University Hospital, Muscat, Oman; ^4^Department of Medicine, Sultan Qaboos University Hospital, Muscat, Oman; ^5^Department of Biochemistry, Sultan Qaboos University Hospital, Muscat, Oman; ^6^Department of Endocrinology, Karolinska University Hospital, Stockholm, Sweden; ^7^Department of Molecular Medicine and Surgery, Karolinska Institute, Stockholm, Sweden

## Abstract

**Background:**

Magnesium (Mg) disorders are common among hospitalized patients and are linked to poor health outcomes. We aimed to determine the incidence of dysmagnesemia among medically hospitalized patients and to identify factors that are associated with dysmagnesemia.

**Methods:**

A prospective cohort study was conducted at Sultan Qaboos University Hospital (SQUH) from April 1st, 2022, to October 31st, 2022, and involved hospitalized adult patients (≥18 years) under the care of the general internal medicine unit. The patients' serum total magnesium (Mg) concentrations were categorized as hypomagnesemia (≤0.69 mmol/L), hypermagnesemia (≥1.01 mmol/L), or dysmagnesemia, which encompassed either hypomagnesemia or hypermagnesemia.

**Results:**

Of the 304 patients evaluated, dysmagnesemia was observed in 22.0%, which comprised of 17.4% with hypomagnesemia and 4.6% with hypermagnesemia. Statistically significant associations were identified between hypermagnesemia and chronic kidney disease (CKD) (*p* = 0.05) and elevated creatinine levels (*p* < 0.01) and lower estimated glomerular filtration rate (eGFR) (*p* < 0.01). Hypomagnesemia was linked to lower ionized calcium (*p* = 0.03) and admission due to infectious diseases (*p* = 0.02). However, ordered regression analysis did not find any significant associations with the different magnesium groups.

**Conclusion:**

Dysmagnesemia was prevalent among hospitalized patients and was associated with different factors; however, ordered regression analysis did not find any association with the different magnesium group, probably due to the limited number of included individuals.

## 1. Introduction

Magnesium (Mg) is a vital element for numerous physiological functions in the human body. It is a cofactor in more than 300 enzymatic reactions required for the structural function of mitochondria, nucleic acid, and proteins [1–3]. In addition, it is an essential mineral for energy molecule metabolism, muscle contraction, and neuromuscular conduction [3]. Studies have shown that Mg can also influence plasma glucose concentration through alteration in insulin stimulation [1, 2]. The majority of Mg is stored in bones, muscles, and soft tissues, while less than 1-2% is found in the blood [4]. In the blood, 55–70% of Mg is in its bioactive ionized form, while 20–30% is bound to proteins, and 5–15% is complexed with anions [4]. The normal reference range for total Mg concentration is between 0.7 and 1.0 mmol/L [5]. Mg disorders are widespread in hospitalized patients, particularly hypomagnesemia, which occurs in 10–24% of them [6, 7]. Hypomagnesemia is more common (65%) in patients admitted to intensive care units [8, 9]. Patients with hypomagnesemia remain asymptomatic until Mg concentration is less than 0.5 mmol/L, and the signs and symptoms include tremors, weakness, and seizures that can lead also to cardiac ischemia and death [10]. Hypomagnesemia has been associated with cancer, alcohol use disorder, and critically ill receiving parenteral nutrition. Also, hypomagnesemia was associated with the use of certain medications such as diuretics and proton pump inhibitors (PPIs) [10].

Hypomagnesemia treatment varies form an immediate and short replacement to a long-term management [11]. While hypermagnesemia is less common with incidence ranging from 5.7% to 23.6%, it has been linked with severe renal impairment and critically ill patients [1, 8, 12, 13]. Hypermagnesemia is a more severe disorder and can be fatal if not treated promptly [14]. Generally, dysmagnesemia has been associated with poor health outcomes, including cardiopulmonary arrest and all-cause mortality [1, 2, 12].

This study aimed to determine the incidence of dysmagnesemia among medically hospitalized adult patients in Oman and to identify factors that were associated with dysmagnesemia.

## 2. Methods

### 2.1. Study Design, Setting, and Population

This was a prospective cohort study conducted at Sultan Qaboos University Hospital (SQUH) from June 8, 2022, to October 25, 2022, involving hospitalized adult patients (≥18 years) under the care of the general internal medicine units [15]. SQUH is an academic tertiary hospital with a capacity of 600 beds and provides multispecialty care for inpatients from all areas of Oman [15].

All patients admitted under the care of general internal medicine unit were screen for inclusion. Exclusion criteria included patients younger than 18 years, those who declined participation, readmission to hospital within 90 days, and situations where obtaining consent was not feasible, such as when a patient lacked the capacity to provide consent and no next of kin was accessible, or if patients left the hospital against medical advice.

### 2.2. Data Collection

Relevant demographic data of patients, including age and gender, as well as medical history (such as hypertension, diabetes mellitus (DM), chronic kidney disease (CKD), and heart failure (HF)), were collected. Medications known to potentially cause dysmagnesemia, such as loop diuretics, thiazides, and proton pump inhibitors (PPIs), were documented. Additionally, certain biochemical data were obtained, encompassing vital electrolyte levels such as ionized calcium, potassium, and sodium, along with albumin level, creatinine level, and estimated glomerular filtration rate (eGFR). Information regarding the treatment of dysmagnesemia and any associated adverse events was recorded. The primary diagnosis was categorized based on the 10th revision of the International Classification of Diseases (ICD10).

The total concentration of Mg was ascertained through a colorimetric end point reaction between magnesium and xylidyl blue in an alkaline solution. This analysis was conducted using the Roche Cobas modular analyzer in the Biochemistry Department at SQUH.

The concentration of ionized calcium was measured using the electrolytes analyzer, Stat Profile Prime Plus® (Nova Biomedicals, Waltham, Massachusetts, USA), which uses the direct ISE method for measurement. The method used to measure serum albumin was based on the specific binding of bromocresol green (BCG) dye. Modification of Diet in Renal Disease (MDRD) formula was used to calculate eGFR (mL/min/1.73 m^2^) = 175 × (serum creatinine) − 1.154 × (Age) − 0.203 × (0.742 if female) × (1.212 if African American).

### 2.3. Definitions

Based on the initial serum total Mg concentration, hypomagnesemia was defined as an Mg concentration of ≤0.69 mmol/L and hypermagnesemia was defined as an Mg concentration of ≥1.01 mmol/L, while dysmagnesemia was defined as any hypomagnesemia or hypermagnesemia.

### 2.4. Ethical Approval

The study was approved by the Medical and Research Ethics Committee at the College of Medicine and Health Sciences, SQU, Muscat, Oman (MREC #2719; SQU-EC/51/2022; dated: April 1^st^, 2022). This research study has been conducted in strict accordance with the ethical guidelines and principles set forth in the Declaration of Helsinki.

#### 2.4.1. Consent

Informed consent was obtained from the patient or his/her next of kin (if capacity was impaired).

### 2.5. Sample Size

The sample size was calculated based on the presumed incidence of dysmagnesemia (combined hypomagnesemia and hypermagnesemia). The previously estimated incidence of dysmagnesemia was 28% among hospitalized patients [8]; we hypothesized that the incidence of dysmagnesemia among our patients will be 25%, with a sample size of 285 patients with a margin error of 5%, and a 95% confidence interval was needed. The sample size was further increased to 300 patients to account for missing follow-up data.

### 2.6. Statistical Analysis

Frequencies and percentages were used to present categorical variables. The median and interquartile ranges (IQRs) were used to describe continuously abnormally distributed variables and mean, and standard deviation were used to describe normally distributed variables. The Kruskal–Wallis test was performed to determine the relationship between abnormally distributed variables and different Mg concentration groups, and one-way ANOVA was used to assess relationship between groups of magnesium and normally distributed variables. The chi-square test was performed to examine the relationships between categorical variables and different Mg concentration groups. When the cells had an anticipated frequency of fewer than five, Fisher's exact test was applied. All relevant characteristics with *p* < 0.2 were included in the backward stepwise regression analysis to identify potential independent factors associated with each group of dysmagnesemia. The two-tailed significance level was established at *p* < 0.05. STATA version 17.0 was used for statistical analysis (StataCorp, 1985–2021, Stata Statistical Software, College Station, TX, USA).

## 3. Results

During the study period, 676 patients were admitted to the general internal medicine unit. Out of these, 304 were included in the study. Exclusions occurred due to the following reasons: being under the age of 18 (*n* = 7), readmission within 90 days (*n* = 72), inadequate blood samples (*n* = 81), refusal to participate (*n* = 150), and inability to obtain consent due to impaired capacity or other logistical reasons (*n* = 62).

In the cohort of 304 patients, 51.0% were female with a median age of 65 years (range 46–76 years). The prevalence of hypomagnesemia was 17.4% (95% CI: 14.3–21.7%), and for hypermagnesemia, it was 4.6% (95% CI: 2.7–7.6%). Overall, the occurrence of dysmagnesemia stood at 22.0% (95% CI: 17.7–27.1%) among adult hospitalized patients.

As depicted in Table 1, elevated creatinine concentrations (*p* < 0.01), reduced eGFR (*p* < 0.01), and the presence of CKD (*p* = 0.05) were more common in the hypermagnesemia group. Conversely, lower ionized calcium concentration was evident in the hypomagnesemia group (*p* = 0.03).

Table 2 displays the primary diagnoses categorized by total Mg concentration groups. Notably, only infectious diseases had a significant association with hypomagnesemia (*p* = 0.02).

The ordered regression analysis did not highlight any statistically significant factors associated with the magnesium groups.

## 4. Discussion

This is one of the few studies that assessed the incidence of hypomagnesemia in hospitalized patients [16] and probably the first study to assess dysmagnesemia in patients hospitalized under general medicine specialty in the Middle East and North Africa (MENA) region [17]. Moreover, it is also one of the few that prospectively included hospitalized patients and not only retrospectively collected data. We showed that the incidence of hypomagnesemia was 17.4%, and the incidence of hypermagnesemia was 4.6%. Furthermore, patients with CKD, lower eGFR, and higher creatinine concentrations were associated with hypermagnesemia.

Previous studies reported the incidence of hypomagnesemia among hospitalized patients ranged between 11.0-20.0% [6, 7], and it was increased to 65.0% in critically ill patients [6, 9]. The observed discrepancies in reported incidences may be attributed to variations in the selection of cutoff values and the diverse clinical characteristics exhibited by the patients. In our study, the incidence of hypomagnesemia is consistent with previously reported incidence of hypomagnesemia. However, the incidence of hypomagnesemia could have been underestimated since only patients admitted to a general medicine ward were included in this study. In contrast, the incidence of hypermagnesemia was similar to previous studies [8], i.e., more uncommon than hypomagnesemia. Hypermagnesemia is described to be extremely rare in patients without renal disease [18, 19]. Moreover, hypermagnesemia is commonly caused by administering Mg-containing medication orally or intravenously [3].

Hypomagnesemia is prevalent among geriatric patients, particularly in critically ill elderly individuals. A study conducted in Nepal aimed to assess the incidence of hypomagnesemia among elderly patients, revealing an incidence of 29.4% among males and 28.5% among females [19]. The study also demonstrated a correlation between hypomagnesemia and adverse outcomes, such as higher mortality rates and increased ventilatory requirements. Hypomagnesemia is primarily attributed to factors such as inadequate Mg intake, impaired intestinal absorption, or medication-induced effects. Interestingly, the median age of patients with hypomagnesemia in the Nepalese study was 70 years, which was similar to the median age observed in the normal and high Mg concentration population [19]. Our study did not demonstrate any link between age and magnesium level.

Hypertension and diabetes mellitus were the most prevalent comorbidities among the current study population, 56.3% and 51.3%, respectively. It is known that both are associated with hypomagnesemia [3, 4]. Nevertheless, no statistically significant association was found with hypomagnesemia in the present study despite the higher incidence compared to other studies, such as a retrospective study in Northern Territory in Australia [9]. Different populations with other ethnicities and fewer included patients in the current study may explain the different results.

We identified CKD in 23.4% of patients, and a correlation was observed between CKD and hypermagnesemia. The regulation of Mg balance primarily relies on intestinal absorption and renal excretion. In moderate CKD, normal serum Mg concentration is maintained by increased fractional excretion of Mg as compensation for loss of renal function. However, this compensatory mechanism becomes ineffective in more advanced diseases (creatinine clearance less than 30 ml/min), and patients on replacement usually develop overt hypermagnesemia [6]. It should be noted that eGFR reflects kidney function better than serum creatinine concentrations since the former often include sex and age in the formula.

While kidney disease can lead to both low and elevated Mg concentrations, our findings indicate that patients with CKD are more likely to develop hypermagnesemia similar to what others have reported [6]. Hypomagnesemia is also common in CKD, especially if the patient is on a diuretic medication [20]. Mg wasting via dialysis is the main reason for hypomagnesemia in end-stage renal disease [20]. Mg supplement was found to have a protective effect in dialysis-dependent patients, and Sakaguchi et al. documented that hypermagnesemia had a survival benefit in ESRD patients on hemodialysis [21].

Multiple medications are associated with hypomagnesemia, e.g., PPIs and diuretics [3]. A systemic review and meta-analysis addressing the relationship between PPIs and hypomagnesemia suggested that PPI use raised the risk of hypomagnesemia by 1.4 folds [22]. In addition, it was mentioned that there are three high risk groups in which the risk of PPIs-induced hypomagnesemia should be considered: those on diuretics, those with low Mg intake, or in patients with GI malabsorption [22]. However, we could not find any correlation between PPIs or diuretics use and hypomagnesemia in the current study.

Hypomagnesemia has been reported in about 30% of patients with alcohol dependence disorders [6], and in our cohort, we identified only 10.2% of patients were alcohol consumers, the latter may be explained due to the study being conducted in a Muslim country. This makes it difficult to establish an association, especially since the amount of alcohol consumed was not collected. Mg is essential in calcium and potassium hemostasis [3]. Our study showed lower levels of ionized calcium levels in the hypomagnesemia group. Existing literature supports the association between hypomagnesemia and hypocalcemia, where low Mg concentrations are believed to impact the release of parathyroid hormone, contributing to a state of hypocalcemia. Furthermore, hypomagnesemia has a negative effect on the renal conversion of 25-hydroxyvitamin D to 1,25-dihydroxy vitamin D [3, 4]. Moreover, calcium and Mg are bound to albumin; hence, any condition that alters albumin levels subsequently alters the levels of both cations. Refractory hypokalemia is another manifestation of hypomagnesemia caused by an excessive increase in renal excretion of potassium. In our study, potassium levels among both the dysmagnesemia group and normomagnesemia were within normal limits.

Emerging data indicate a potential relationship between Mg and immune system functions. Mg is integral to various immune processes, including immunoglobulin creation, immune cell attachment, and antibody-driven cytolysis [23]. The current study demonstrated an association between hypomagnesemia and admission due to infectious diseases. Hypomagnesemia was linked to increase risk of infection in renal transplant recipients [23], and poor outcomes in critically sick patients with sepsis [24]. However, it should be noted that ordered regression analysis with several factors did not find any significant associations with the different magnesium groups. The reason may be the limited number of included patients.

This study has some limitations including the study being conducted at a single center, which affects its generalizability. Additionally, data regarding regular Mg supplements and information on follow-up Mg concentration were not captured. Probably, the study was underpowered to study association between magnesium disorders and common medical patients' characteristics and medications. However, this study was a prospective study instead of the usual retrospective nature of most previously published studies in the field [3]. Thus, our study lacks many of the biases common in retrospective studies.

## 5. Conclusion

Dysmagnesemia was common among adult patients hospitalized in medical wards. CKD and a lower eGFR were associated with hypermagnesemia, while infectious diseases and decreased ionized calcium levels were linked to hypomagnesemia. However, the ordered regression analysis with several factors did not find any significant associations with different magnesium groups. Further studies with larger sample sizes are needed to understand the significance of magnesium disorders in medically hospitalized patients.

## Figures and Tables

**Table 1:** Characteristics of 304 hospitalized patients according to total serum magnesium concentration.

Characteristic *n* (%) unless specified otherwise	Total 304 (100%)	≤0.69 mmol/L 53 (17.4%)	0.70–1.00 mmol/L 237 (78.0%)	≥1.01 mmol/L 14 (4.6%)	*p* value
Gender (female)	155 (51.0%)	28 (53.0%)	116 (49.0%)	11 (78.6%)	0.09
Age; IQR, years	65 (46–76)	70 (61–76%)	64 (45–76)	69 (45–82)	0.06
*Medical history*					
Hypertension	171 (56.2%)	33 (62.3%)	127 (53.6%)	11 (78.6%)	0.12
Diabetes mellitus (DM)	156 (23.4%)	27 (18.9%)	120 (22.8%)	9 (50.0%)	0.61
Chronic kidney disease (CKD)	71 (23.4%)	10 (22.7%)	54 (23.2%)	7 (28.6%)	0.05
Heart failure (HF)	71 (23.4%)	12 (22.6%)	55 (23.2%)	4 (28.6%)	0.89
Atrial fibrillation (AF)	24 (7.9%)	3 (5.7%)	18 (7.6%)	3 (21.4%)	0.14
Alcohol consumption	31 (10.2%)	7 (13.2%)	24 (10.1%)	0	0.42
Smoking	41 (13.5%)	11 (20.8%)	29 (12.2%)	1 (7.1%)	0.24
*Medications*					
Loop diuretics	100 (32.9%)	19 (35.9%)	74 (31.2%)	7 (50.0%)	0.31
Thiazides	17 (5.6%)	4 (7.55%)	13 (5.51%)	0	0.60
Proton pump inhibitors (PPI)	155 (51.0%)	31 (58.5%)	117 (49.4%)	7 (50.0%)	0.49
*Biochemical profile*					
Albumin, SD, g/L	35.57 ± 7.05	34.0 ± 7.9	36.1 ± 7.0	36.6 ± 8.8	0.13
Ionized calcium, IQR, mmol/L	1.20(1.16–1.24)	1.18(1.14–1.22)	1.21(1.17–1.24)	1.22(1.17–1.25)	0.03
Sodium, IQR, mmol/L	136 (123–139)	135 (130–137)	136 (132–139)	137 (133–142)	0.09
Potassium, IQR, mmol/L	4.3 (3.9–4.7)	4.4 (3.9–4.8)	4.2 (3.9–4.65)	4.65 (4.2–5.1)	0.09
Serum creatinine, IQR, mmol/L	78 (57–119.5)	76 (60–115)	77 (56–114)	169 (86–370)	<0.001
eGFR	84(57–90)	84 (63–90)	85(60–90)	27(13–57)	0.001
*Dysmagnesemia treatment*					
Oral or IV Mg	59 (19.4%)	24 (45.3%)	33 (13.9%)	2 (14.3%)	<0.001

**Table 2:** Primary diagnosis according to ICD10 according to total serum magnesium concentration in 304 hospitalized patients.

Primary diagnosis (ICD-10)	Total 304 (100%)	≤0.69 mmol/L 53 (17.4%)	0.70–1.00 mmol/L 237 (78.0%)	≥1.01 mmol/L 14 (4.6%)	*p* value
Infectious disease (A00–B99)	40 (13.2%)	13 (24.5%)	27 (11.4%)	0	0.02
Endocrine, nutritional, and metabolic diseases (E00–E99)	23 (7.6%)	4 (7.6%)	18 (7.6%)	1 (7.1%)	1.00
Diseases of the nervous system (G03–G72)	10 (3.3%)	2 (3.8%)	8 (3.4%)	0	1.00
Diseases of the circulatory system (I16–I99)	79 (26.0%)	11 (20.8%)	65 (27.4%)	3 (21.4%)	0.58
Diseases of the respiratory system (J00–J99)	58 (19.1%)	9 (17.0%)	45 (19.0%)	4 (28.6%)	0.58
Diseases of the digestive system (K00–K93)	31 (10.2%)	7 (13.2%)	21 (8.9%)	3 (21.4%)	0.16
Diseases of the genitourinary system (N00–N99)	22 (7.2%)	2 (3.8%)	19 (8.0%)	1 (7.2%)	0.55
Others	41 (13.5%)	5 (9.4%)	34 (14.4%)	2 (14.3%)	0.710

## Data Availability

The data are not accessible to readers.
